# Effectiveness of antenatal dexamethasone in reducing respiratory distress syndrome and mortality in preterm neonates: a nested case control study

**DOI:** 10.1186/s12887-023-03887-5

**Published:** 2023-03-01

**Authors:** Wema Kibanga, Ritah F. Mutagonda, Robert Moshiro, Alphonce Mareale, Manase Kilonzi, Hamu J. Mlyuka, Wigilya P. Mikomangwa, Peter P. Kunambi, Appolinary Kamuhabwa, Omary Mashiku Minzi

**Affiliations:** 1 School of Pharmacy, Muhimbili University of Health and Allied Sciences, P.O. Box 65001, Dar Es Salaam, Tanzania; 2 Muhimbili National Hospital, P.O. Box 65000, Dar Es Salaam, Tanzania; 3 School of Medicine, Muhimbili University of Health and Allied Sciences, P.O. Box 65001, Dar Es Salaam, Tanzania

**Keywords:** Antenatal dexamethasone, Respiratory distress syndrome, Preterm neonates

## Abstract

**Background:**

Respiratory distress syndrome (RDS) is a significant cause of preterm neonatal morbidity and mortality globally. Measures like the use of antenatal corticosteroids (ACS) and immediate resuscitation of the newborn after birth are taken to abate preterm related complications. Most studies that evidenced the benefit of ACS were done in high resource settings. Therefore, this study was conducted to assess the effectiveness of ACS in reducing RDS and neonatal mortality in preterm neonates in resource-limited settings.

**Methods:**

A three months prospective nested case–control study (1:2 unmatched) was conducted at Muhimbili National Hospital and Amana regional referral hospital in Dar es salaam, Tanzania. Neonates delivered at 28 to 34 gestational weeks were enrolled and followed up until discharge. Data analysis was done using the statistical package of social sciences version 23. Logistic regression analysis was used to determine the effect of ACS on the RDS and mortality in the cohort, controlling for important maternal and neonatal variables. All tests were considered statistically significant at *p* < 0.05.

**Results:**

Out of 330 preterm neonates enrolled, 110 were cases and 220 were controls. The median gestational age at delivery was 30 weeks and 6 days (IQR 4.68) among cases and 33 weeks (IQR 3) among controls. One-minute APGAR score of < 7 (AOR: 3.11; 95% CI 1.54–6.30), and neonatal birth weight (AOR: 0.998; 95% CI 0.997–0.999) were significantly associated with RDS. No significant association was observed between ACS exposure and RDS occurrence (AOR: 1.65; 95% CI 0.86 – 3.15). The overall mortality rate was 9 per 1000 neonates. Neonatal mortality occurred only among cases whereby, a unit increase in gestational age was associated with a 30% reduction in neonatal mortality (Adjusted hazard ratio, AHR: 0.70, 95% CI: 0.5–0.92, *p* = 0.011).

**Conclusion:**

Decrease in gestational age, one minute APGAR score of < 7 and decreasing birth weight were associated with RDS among preterm neonates. ACS was not associated with reduced RDS occurrence and neonatal mortality rates. Moreover, increase in gestation age was the only factor found to be protective against preterm neonatal mortality.

## Introduction

Preterm delivery, defined as delivery before completion of 37 weeks of gestation, is the leading cause of perinatal and neonatal mortality and morbidity [1]. Preterm birth is a global problem; however, 81% of all preterm births occur in Asia and sub-Saharan Africa [2]. Due to their immaturity, preterm infants are vulnerable to several complications such as feeding difficulties, impaired thermoregulation, short and long term neurological problems.

Respiratory distress syndrome (RDS), also known as hyaline membrane disease, is a breathing disorder commonly affecting premature infants because their lungs are not able to make enough surfactant [3]. Factors like infections and smoking during pregnancy, gestational diabetes, type of delivery and stress during delivery increase the likelihood of RDS in newborns [4]. Several measures are in place to improve newborn outcomes in the settings of premature delivery, including the administration of antenatal corticosteroids (ACS).

The World Health Organisation (WHO) recommends using either dexamethasone or betamethasone for women at risk of preterm birth from 24 to 34 weeks of gestation when preterm birth is considered imminent, with no clinical evidence of maternal infection and adequate childbirth care is available [5, 6]. An optimum time of delivery from corticosteroid administration is 2 to 7 days. A single repeat course of ACS is recommended if preterm birth does not occur within 7 days after the initial dose, and a subsequent clinical assessment demonstrates a high risk of preterm birth in the next 7 days [5].

In Tanzania, prematurity and its complications are among the leading causes of death among neonates [7]. Since 2015, dexamethasone has been widely available to the district hospital level following the preparation of national guidelines for ACS use and their addition to the national essential medicine list. However, there have been a slow decline in neonatal mortality from 22.1 deaths per 1000 live birth in 2015 to 20.3 deaths per 1000 live births in 2019 [8]. Tanzania has limited facilities that can manage preterm neonates with any respiratory distress since the capacity to deliver continuous positive airway pressure (CPAP), intubate and administer surfactant as indicated is inadequate [9].

The Cochrane meta-analysis of 2017 that forms a basis of WHO recommendations; included 29 trials conducted in high resource countries with standard neonatal care [10]. Another review on the impact of ACS in low and middle-income countries had inconclusive findings because some studies in such settings reported no benefit of ACS in reducing neonatal mortality and RDS [11]. The ACTION trial that included a total of 2852 women (and their 3070 fetuses) across tertiary hospitals in Bangladesh, India, Kenya, Nigeria, and Pakistan who were at risk for early preterm birth reported that the use of dexamethasone resulted in significantly lower risks of neonatal death alone and stillbirth or neonatal death than the use of placebo, without an increase in the incidence of possible maternal bacterial infection [12]. Thus, there is a need for more evidenceon the benefit of ACS use in low resource settings where pregnant women start antenatal visits late together with inadequate number of neonatal care facilities.

This study, therefore, aimed at assessing the effectiveness of ACS in reducing RDS and neonatal mortality in preterm neonates in resource-limited settings.

## Methods

### Study design and settings

This was a three months hospital based prospective nested case control (1:2 unmatched) study conducted from March to May 2021 in two Dar es Salaam referral hospitals including Muhimbili National Hospital (MNH) and Amana regional referral hospital. This was unmatched study, however cases and controls were time censored. The two study sites are equipped with neonatal care units with machinery and trained personnel to provide Continuous positive airway pressure (CPAP), thermoregulation, mechanical ventilation consintently,thus ensuring good care is given to the neonates.

### Study population

Neonates delivered between 28 to 34 weeks of gestation.

### Inclusion criteria

All neonates admitted to Neonatal Care Unit (NCU), whose mothers provided written consents on their inclusion in the study. Neonates were recruited within 24 h after birth. RDS was diagnosed by clinicians through observing the presence of respiratory rate (RR) > 60/minute, subcostal or intercostal recessions, expiratory grunt or groaning, presence of nasal flaring, suprasternal retractions, decreased air entry on auscultation of the chest, gasping, choking and presence of cyanosis. Silverman Anderson Score was used in confirming and assessing severity of RDS [13].

### Exclusion criteria

Neonates delivered below 28 weeks of gestation and beyond 34 weeks of gestation**,** neonates diagnosed with hypoxic ischemic encephalopathy (based on Thompson score) or a primary neuromuscular condition and those with any congenital anomaly. Gestational age determination was done by considering ultrasound measurement of the embryo in the first trimester, fundal height and details of last menstruation. Ballard’s score assessed by clinicians was used to confirm the neonates gestation age within 24 h of birth [14].

### Sample size

Sample size was calculated using Kelsey’s formula for unmatched case control studies sample size calculation [15]. The calculation employed two proportions from a study in Pakistan which reported 26% proportion of controls with exposure, 12.5% proportion of cases with exposure [16]. Therefore, sample size of (*N* = 330) was used in this study including 110 cases and 220 controls.

### Data collection

A data abstraction form consisting of three main sections namely neonatal birth history, maternal obstetrics history including maternal comorbidities and neonatal clinical history including neonatal outcomes was pretested using 16 subjects prior to adoption for data collection. Data was collected by the principal investigator and three research assistants. Data was obtained from patient files, registries, pharmacy data base as well as antenatal clinic cards, on data was obtained from interview with the mothers. Enrolled neonates were followed up throughout the study period while being monitored for oxygen saturation, medications used and mode and duration of oxygen supplementation.

### Data analysis

Data from the data abstraction forms were entered in excel sheets and then exported to and coded using Statistical Package for Social Sciences (SPSS) version 23. Descriptive statistics were summarized using proportions. Chi-square/Fisher’s test and logistic regression was used to test the descriptive statistics and establish association between categorical variables respectively. Continuous variables were analyzed using Mann Whitney test where the descriptive part was summarized using median and range. Univariate and multivariate binary logistic regression analysis was employed to determine the effect of ACS on the RDS and mortality in the cohort, controlling for important maternal and neonatal variables. The overall mortality rate was calculated using incidence rate.. Log ranking test and cox regression were used to graphically compare probability of death with time and measure association, respectively. A *p*-value of < 0.05 was considered statistically significant at 95% confidence interval.

## Results

### Baseline neonatal and maternal characteristics of cases and controls

A total of 330 preterm neonates enrolled, of which 110 were cases and 220 were controls. 71.8% of the participants were from MNH and 28.2% from Amana regional referral hospital. The median gestational age at delivery was 30 weeks and 6 days (28-34) among cases and 33 weeks (28-34) among controls (*p* < 0.001). A one-minute Apgar score of < 7 was assigned to 38.2% of cases compared to 14.5% of controls (*p* < 0.001). The median birth weight was 1400 (600–2400) among cases and 1800 (800–2700) among controls (*p* < 0.001). (Table 1 and Table 2).

**Table 1:** Baseline neonatal characteristics (n = 330)

Variable	Case *n* = 110 (%)	Control *n* = 220 (%)	*P*-Value
Gender of baby			0.273
Male	65 (59.1)	116 (52.7)	
Female	45 (40.9)	104 (47.3)	
1 min APGAR score			**< 0.001**
< 7	42 (38.2)	32 (14.5)	
≥ 7	68 (61.8)	188 (85.5)	
5 min APGAR score			**< 0.001**
< 7	13 (11.8)	4 (1.8)	
≥ 7	97 (88.2)	216 (98.2)	
Birth weight			**< 0.001**
Median	1400	1800	
IQR	540	600	
Gestational age at delivery			**< 0.001**
Median	30.8571	33	
IQR	4.68	3

**Table 2:** Baseline maternal characteristics (*n* = 330)

Variable	Case *n* = 110 (%)	Control *n* = 220 (%)	*p*-value
Maternal age at delivery (years)			
Median	28	27	0.520
IQR	8	9	
Marital status			
Married	91 (82.7)	160 (72.7)	**0.045**
Not married	19 (17.3)	60 (27.3)	
Level of education			
Informal	6 (5.5)	24 (10.9)	0.144
Primary	42 (38.2)	57 (25.9)	
Secondary	46 (41.8)	99 (45.0)	
College	4 (3.6)	11 (5.0)	
University	12 (10.9)	29 (13.2)	
Occupation			
Employed	11 (10.0)	21 (9.5)	0.959
House wife	42 (38.2)	79 (35.9)	
Self employed	46 (41.8)	99 (45.0)	
Unemployed	11 (10.0)	21 (9.5)	
Mode of delivery			
SVD	72 (65.5)	126 (57.3)	0.153
Caesarean section	38 (34.5)	94 (42.7)	
Gravidity			
Primigravida	36 (32.7)	88 (40.0)	0.188
Multigravida	74 (67.3)	132 (60.0)	
Prenatal care			
Received	102 (92.7)	212 (96.4)	0.094
Not received	8 (7.3)	8 (3.6)	
Maternal NCDs			
Yes	50(45.5)	116 (52.7)	1.000
No	60(54.5)	104 (47.3)	
PPROM			
Yes	42 (38.2)	78 (35.5)	0.627
No	68 (61.8)	142 (64.5)	

### Effect of ACS

**Fig. 1:**
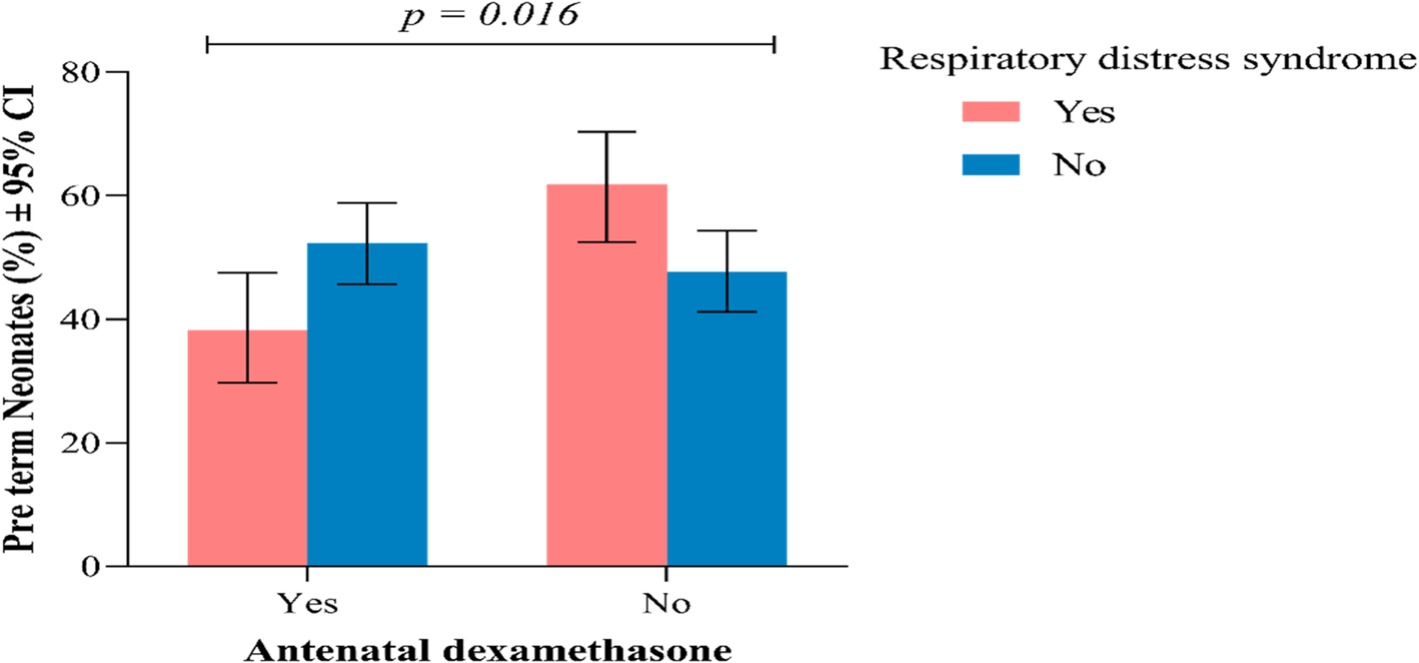
Proportion of preterm neonates with respect to antenatal dexamethasone exposure and RDS

RDS was found to occur more among preterm neonates whose mothers did not receive ACS compared to those who received ACS (61.8% with 95% CI (52.5—70.4)) vs (38.2% with 95% CI (29.6—47.5), *p* = *0.016* (Fig. 1).

### Predictors of respiratory distress syndrome

Gestational age at delivery (AOR: 0.81; 95% CI 0.69–0.94), 1 min Apgar score of less than 7 (AOR: 3.11; 95% CI 1.54–6.30), and neonatal birth weight (AOR: 0.998; 95% CI 0.997–0.999) were significantly associated with RDS**.** Hereby, a complete week increase in gestational age at delivery is associated with a 19% decrease in the odds of developing RDS whereas a gram increase in neonatal birth weight is associated with a 0.2% reduction in RDS risk. Neonates who scored a one-minute Apgar score of < **7** were three times more likely to be diagnosed with RDS compared to those with APGAR score ≥ 7. ACS exposure was not associated with RDS (AOR: 0.61; 95% CI 0.86–3.15, *p* = *0.129)* as summarized in Table 3.

**Table 3:** Univariate and Multivariate analysis of factors associated with RDS

	**Univariate analysis**			**Multivariate analysis**		
**Variable**	**COR**	**95% CI**	***p* -value**	**AOR**	**95% CI**	***p*—value**
Gestational age (weeks)	0.65	0.57 – 0.74	**< 0.001**	0.81	0.69 – 0.94	**0.007**
Apgar score (1 min)						
< 7	3.63	2.12 – 6.21	**< 0.001**	3.11	1.54 – 6.30	**0.002**
≥ 7	Ref					
Apgar score (5 min)						
< 7	7.24	2.30 – 22.76	**< 0.001**	2.19	0.52 – 9.28	0.287
≥ 7	Ref					
Mode of delivery						
Caesarean	0.71	0.44 – 1.14	0.153	1.23	0.63 – 2.36	0.562
NSVD	Ref					
Marital status						
Unmarried	0.56	0.31 – 0.99	**0.047**	0.54	0.5 – 1.19	0.125
Married	Ref					
Level of education						
Primary	2.95	1.11 – 7.85	**0.031**	2.87	0.87 – 9.54	0.085
Secondary	1.86	0.71 – 4.86	0.206	2.08	0.64 – 6.79	0.227
College	1.46	0.34 – 6.22	0.613	1.44	0.26 – 8.15	0.680
University	1.66	0.54 – 5.07	0.378	2.55	0.64 – 10.22	0.186
Informal	Ref					
Gravida						
Multigravida	1.38	0.85 – 2.23	0.189	0.94	0.49 – 1.78	0.844
Primigravida	Ref					
Prenatal care						
Yes	0.42	0.15 – 1.19	0.104	0.53	0.15 – 1.88	0.322
No	Ref					
ANC						
Yes	1.77	1.11 – 2.83	**0.016**	1.65	0.86 – 3.15	0.129
No	Ref					
Birth weight in (gm)	0.997	0.997 – 0.998	**< 0.001**	0.998	0.997 – 0.999	**< 0.001**

Key: *COR* Crude odds ratio, *AOR* Adjusted odds ratio, *Ref* Reference group, NSVD:..,ANC:

### Preterm neonatal mortality

The overall mortality rate was found to be 9 per 1000 neonates. Neonates delivered by mothers who did not receive ACS had a high mortality rate (13 per 1000 neonates) compared to those whose mothers received antenatal dexamethasone (4 per 1000 neonates). The highest mortality rate was observed in neonates diagnosed with RDS. No deaths occurred in neonates without RDS. Neonates with extremely low birth weight had a high mortality rate (31 per 1000 neonates) compared to other birth weight categories. Neonates with a one-minute and five-minute Apgar scores of < **7**had high mortality rates compared to scores greater than or equal to seven (14 per 1000 neonates and 18 per 1000 neonates respectively). Neonates delivered by mothers with non-communicable comorbidities had the lowest mortality rates (8 per 1000 neonates) compared to those whose mothers had no comorbidities. Neonates with gestational age of less than 32 weeks at delivery had a high mortality rate (15 per 1000 neonates) compared to those with 32 weeks and above. Primigravids and those delivered by spontaneous vaginal delivery had higher mortality rates compared to multigravidas and caesarean Sect. (12 and 11 per 1000 neonates), respectively (Table 4).

**Table 4:** Mortality rate stratified by socio-demographic and clinical characteristics of the study participants

Characteristic	Category	Total N	Person time (Days)	Death N (%)	Incidence rate /1000 Neonates
RDS	Yes	110	1,052	23 (20.9)	22
	No	220	1,420	0 (0)	0
ANC	Yes	157	1,057	4 (2.5)	4
	No	173	1,415	19 (11)	13
Maternal NCDs	No	164	1,172	13 (7.9)	11
	Yes	166	1,300	10 (6.0)	8
Gravidity	Primigravida	124	815	10 (8.1)	12
	Multigravida	206	1,653	13 (6.3)	8
Birth weight	< 1000 g	17	192	6 (35.3)	31
	1000-1499 g	86	800	10 (11.6)	13
	1500-2499 g	221	1,458	7 (3.2)	5
	≥ 2500 g	6	22	0 (0)	0
1 min APGAR score	< 7	74	643	9 (12.2)	14
	≥ 7	256	1,829	14 (5.5)	8
5 min APGAR score	< 7	17	164	3 (17.6)	18
	≥ 7	313	2,308	20 (6.4)	9
Mode of delivery	SVD	198	1,519	17 (8.6)	11
	Caesarean section	132	953	6 (4.5)	6
Gestational age at delivery	< 32	125	1,176	18 (14.4)	15
	32–34	205	1,296	5 (2.4)	4

^*^The overall mortality rate is 0.0093042 (9/1000 neonates per day)

### Factors associated with mortality

**Fig. 2:**
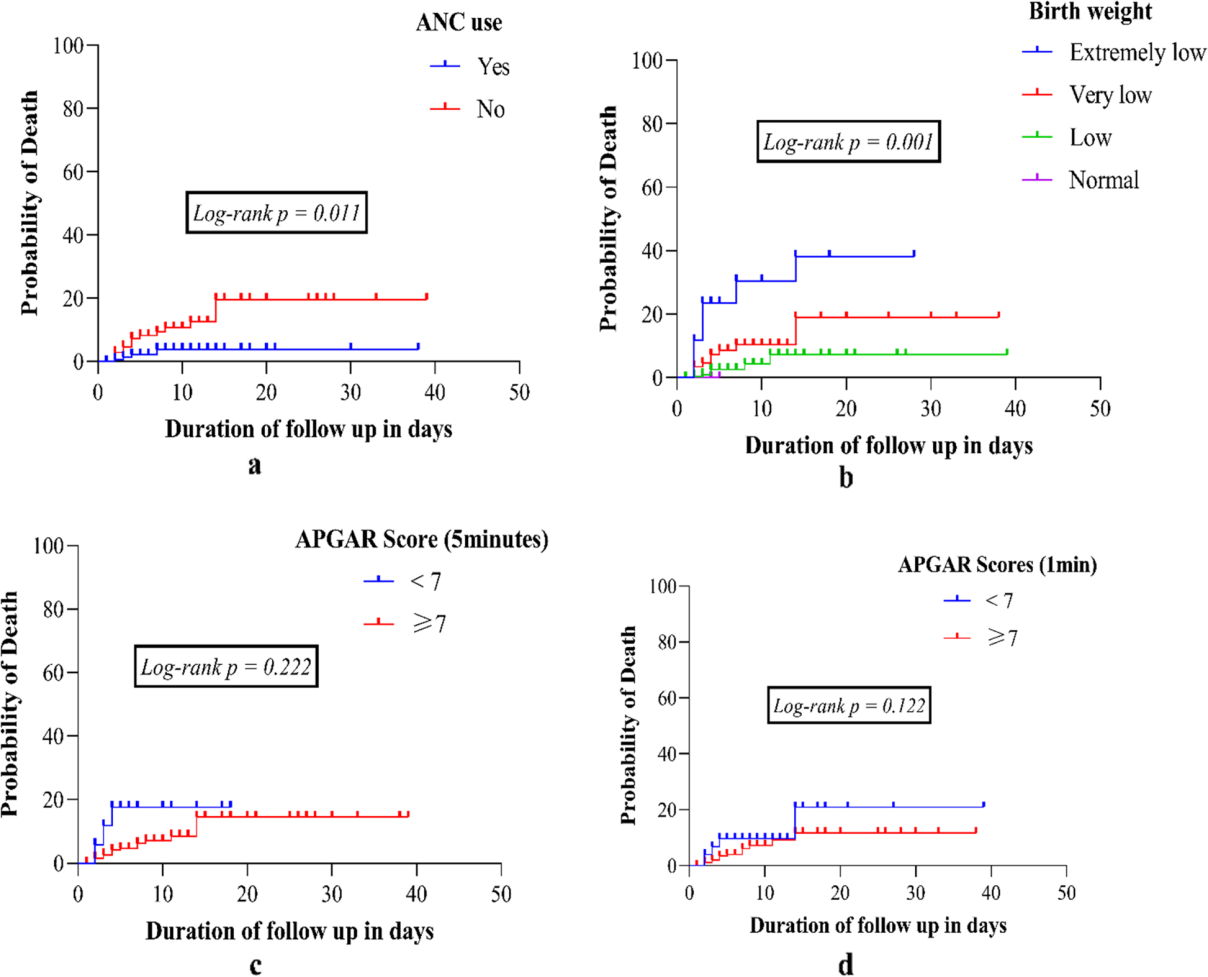
Cumulative hazard curves showing the association between probability of death and time using Log-ranking test

Log rank test revealed that there is a significantly increased rate of death in neonates not exposed to antenatal dexamethasone (Fig. 2a, p = 0.011), extremely low birth weight (Fig. 2b, p = 0.001), a 5-min APGAR score of < 7(Fig. 2c, p = 0.222) and a 1-min APGAR score of < **7**(Fig. 2d, p = 0.122).

**Fig. 3:**
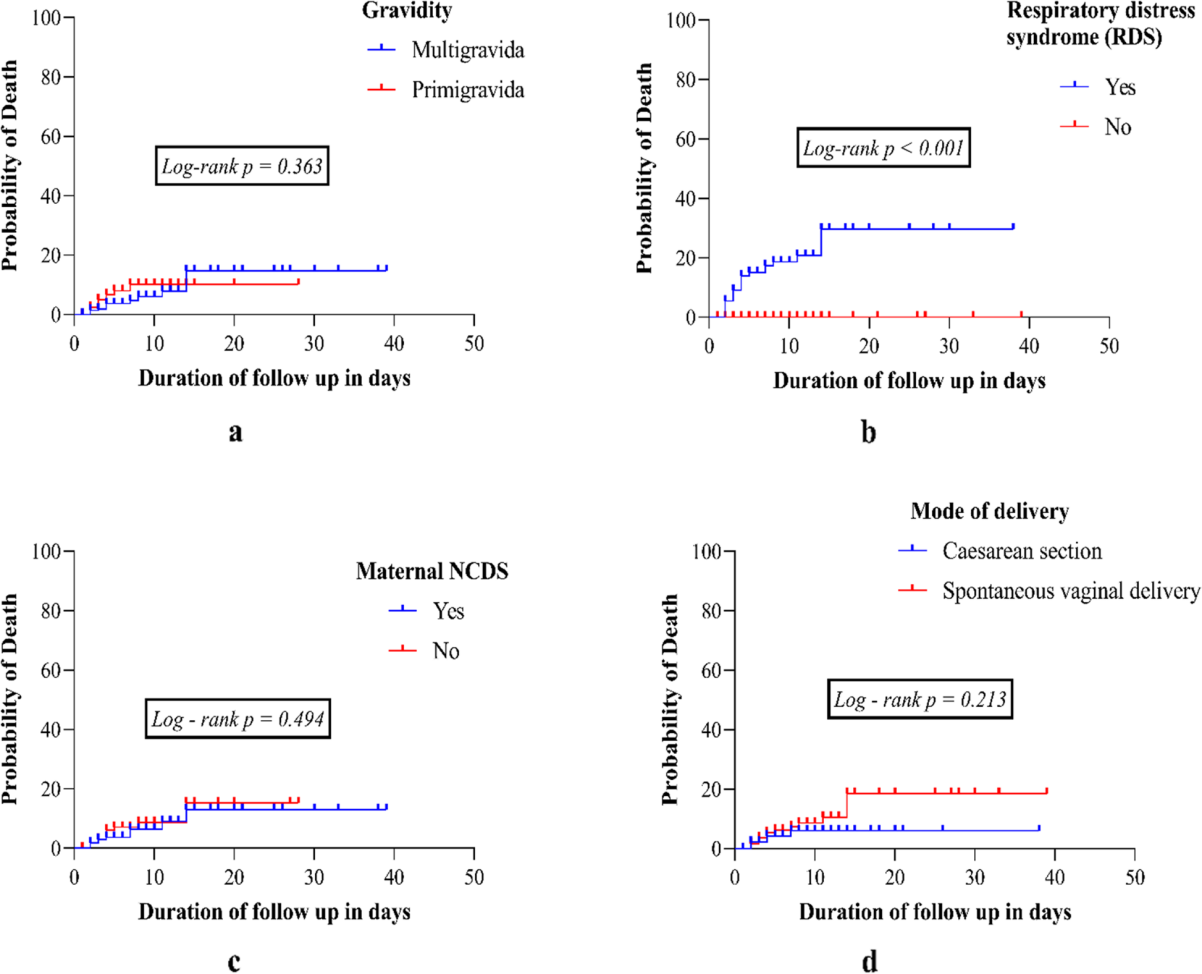
Cumulative hazard curves showing the association between probability of death and time using Log-ranking test

A greater rate of death was observed in neonates with RDS (Fig. 3b, P < 0.001), however no significant association was found with other factors including; primigravida, neonates whose mothers had no comorbidities and those delivered by spontaneous vaginal delivery (Fig. 3a, 3c, 3d respectively).

Further analysis using Cox-regression demonstrated that only gestational age was significantly associated with mortality. A unit increase in gestational age had a 30% decrease in the risk of death (AHR: 0.70, 95% CI: 0.53–0.92, *p* = 0.011) (Table 5). Although univariate analysis found an association between mortality and antenatal dexamethasone exposure (CHR: 0.27, 95% CI: 0.09–0.80, *p* = 0.018), birth weight (CHR: 0.99, 95% CI: 0.997–0.999, *p* < 0.001) and RDS (CHR: 0.01, 95% CI: 0.01–0.29, *p* = 0.008); this was not replicated in multivariate analysis.

**Table 5:** Univariate and multivariate Cox regression analysis for the risk factors for mortality among preterm neonates

	**Univariate analysis**			**Multivariate analysis**		
**Variable**	**CHR**	**95% CI**	***P*—Value**	**AHR**	**95% CI**	***P*—Value**
Use of ACS						
Yes	0.27	0.09 – 0.80	**0.018**	0.41	0.13 – 1.26	0.118
No	Ref					
Birth weight	0.99	0.997 – 0.999	**< 0.001**	0.999	0.998 – 1.00	0.131
APGAR Score (1 min)						
< 7	1.91	0.82 – 4.42	**0.132**	1.46	0.61 – 3.47	0.397
≥ 7	Ref					
APGAR Score (5 min)						
< 7	2.09	0.62 – 7.11	0.236			
≥ 7	Ref					
Gravidity						
Primagravida	1.46	0.64 – 3.35	0.369			
Multigravida	Ref					
RDS*						
No	0.01	< 0.01 – 0.29	**0.008**			
Yes	Ref					
Maternal NCDs						
Yes	0.75	0.33 – 1.72	0.499			
No	Ref					
Mode of delivery						
Caesarean	0.56	0.22 – 1.42	0.223			
SVD	Ref					
Gestational age	0.59	0.47 – 0.76	**< 0.001**	0.70	0.53 – 0.92	**0.011**

Keys: *CHR* Crude hazard ratio, *AHR* Adjusted hazard ratio

## Discussion

This study found no association between ACS use and RDS occurrence. This is contrary to other studies for example a study conducted in Brazil whereby RDS incidence was reduced with antenatal corticosteroid use [17]. Differences in the epidemiology of preterm birth, exposure to infections, pharmacogenetical factors and quality of neonatal care could be the likely reasons for the observed differences.

Gestational age at delivery and neonatal birth weight were found to be independent predictors of RDS in preterm neonates. Hereby, a complete week increase in gestational age at delivery is associated with a 19% decrease in the odds of developing RDS where as a gram increase in neonatal birth weight is associated with a 0.2% reduction in RDS odds. There is a direct relationship between gestational age at delivery and neonatal birth weight [18]. These two are directly related to RDS occurrence because the latter happens as a result of immature lungs with inadequate surfactant. Surfactant production in the fetus mostly occurs after 30 weeks of gestation. Thus, neonates born prior to this age are likely to suffer from RDS [19]. These findings are similar to a study conducted in the neonatal unit in Cameroon to assess the prevalence, predictors and outcomes of neonatal distress [20]. Several other studies have reported gestational age and neonatal birth weight as predictors of RDS in preterm neonates [21, 22]. Also, in this study, 1 min Apgar score of less than 7 was found to be an independent predictor of RDS in preterm neonates. Hereby preterm neonates who scored less than 7 at one minute were three times more likely to develop RDS compared to those with an APGAR score of more than 7. This finding is similar to the findings of a prospective study in Ethiopia that showed an APGAR score less than 7 (AHR: 3.1 (95%CI: 1.8–5.0) to be a significant predictor of RDS in preterm neonates [23]. Apgar score is used as a quick assessment tool of neonates immediately after birth. Therefore, neonates with immature lungs might struggle to initiate and maintain spontaneous respiration after birth.

This study found an overall mortality rate of 9 per 1000 preterm neonates. Death was reported in 23 (6.97%) of the preterm neonates. In this study, not receiving ACS, low birth weights, RDS, low APGAR scores and prematurity have been associated with increased mortality rates. This could be due to the fact that preterm newborns are at greater risk of death, which could result from physical and physiologic immaturities [24]. These findings are consistent with other studies and reports in sub Saharan Africa [25]. This can be because many countries in this region do not have enough facilities in terms of expertise and equipment that are capable of providing adequate care to neonates with respiratory problems and those who suffered from intrapartum hypoxia. However, contrary to our study, maternal comorbidities were associated with increased neonatal mortality rates [25]. In our study neonates delivered by mothers with non-communicable comorbidities had the lowest mortality rates (8 per 1000 neonates) compared to those whose mothers had no comorbidities. This could be because mothers with comorbidities are more likely to receive preventive care as the likelihood of preterm delivery is expected compared to those without.

Another study conducted at a neonatal unit in northern Tanzania on cause of specific neonatal mortality documented that prematurity was the second leading single cause of neonatal deaths especially in low birth weight neonates (94.7%), whereby case fatality declined with increasing birth weight [26]. This is similar to our findings whereby more deaths occurred in the extremely low birth weight neonates and no deaths occurred in normal birth weight ones. Several other studies conducted in Tanzania report similar findings [27, 28].

In our study fewer deaths occurred in the neonates exposed to antenatal dexamethasone (2.5%) compared to those unexposed to antenatal dexamethasone (11%). Although antenatal dexamethasone decreased incidence of neonatal mortality, there was no significant association upon multivariate analysis. These findings are consistent with several other studies that have shown fewer deaths with dexamethasone use [17, 29, 30]. These findings are also consistent with those reported by the ACTION trial that included a total of 2852 women (and their 3070 fetuses) across tertiary hospitals in low and middle income countries who were at risk for early preterm birth whereby the use of dexamethasone resulted in significantly lower risks of neonatal death alone and stillbirth or neonatal death than the use of placebo, without an increase in the incidence of possible maternal bacterial infection [12]. However, these findings are contrary to the antenatal corticosteroid trial (ACT trial) done in low resource settings, which found an overall increase in 28-day neonatal mortality and stillbirth associated with the intervention[31], and a study in a rural Tanzanian hospital, which found poor neonatal outcomes associated with antenatal corticosteroid use [32]. This could be because our study was conducted in a tertiary and referral hospital which have the required facilities and expertise to care for preterm neonates.

In this study, majority of the deaths occurred in neonates delivered at 28 to 32 gestational weeks (14.4%). Similar to our findings, a multicenter hospital-based investigation of preterm neonatal mortality in China found most of the deaths (57.9%) occurred in neonates delivered at 28 to 32 weeks of gestation [33]. A unit increase in gestational age was independently associated with a 30% reduction in neonatal mortality risk, according to our findings. Furthermore, in this study, spontaneous vaginal delivery had higher mortality rates compared to caesarean section. These findings are consistent to a study conducted in the US on very preterm infants, comparing the mortality rates between neonates delivered by primary spontaneous vaginal delivery and caesarean section. The study found that caesarean section significantly reduced the risk of neonatal death among the very preterm neonates (adjusted odds ratios of 0.58, 0.52, 0.72, and 0.81 for 22, 23, 24, and 25 weeks, respectively) [34]. However, contrary to our findings, a review on trends in neonatal, post-neonatal, infant, child and under-five mortalities in Tanzania from 2004 to 2016 showed that neonates delivered by caesarean section had a higher risk of mortality compared to vaginal delivery [35]. This can be because the study was conducted in referral hospitals with enough resources and well-trained personnel.

### Limitations

Standard diagnosis of RDS includes performing chest X-ray, however this was not routinely done to all neonates with RDS. Also, this study could be limited by the general limitation of nested case control studies; that is, reduced precision and power due to sampling of controls. However, the findings in this study are acceptable despite the limitations because of the mitigation measures employed like time censoring cases and controls as well as confirmation of RDS diagnosis using Silverman Anderson Scores performed by well-trained clinicians.

## Conclusion

Increase in gestational age was found to be protective against neonatal mortality and RDS. One minute APGAR score of < 7 and low neonatal birth weights are predictors of RDS in preterm neonates. ACS was not associated with reduced neonatal mortality rates and RDS occurrence in preterm neonates. Larger prospective studies should be conducted to determine the exact preconditions of antenatal corticosteroid therapy in low-resource settings.

## Data Availability

The data sets used and/or analysed during the current study are available from the corresponding author on reasonable request.

## Abbreviations

ACS: Antenatal corticosteroids
AHR: Adjusted hazard ratio
AIP: Abnormally invasive placentation
APGAR: Appearance, Pulse, Grimace, Activity and Respiration
BMI: Body mass index
CHR: Crude hazard ratio
IVH: Intraventricular hemorrhage
LMICs: Low and middle income countries
MNH: Muhimbili national hospital
NEC: Necrotizing enterocolitis
NCU: Neonatal care unit
PROM: Premature rupture of membranes
RDS: Respiratory distress syndrome
RNA: Ribonucleic acid
SPSS: Statistical package for social sciences
